# Plasma Glial Fibrillary Acidic Protein and Neurofilament Light Chain Concentrations Are Inversely Associated with Retinal Microvascular Perfusion and Vessel Density in Cognitively Normal Individuals with Familial or Genetic Risk Factors for Alzheimer’s Disease

**DOI:** 10.3390/diagnostics16172764

**Published:** 2026-08-28

**Authors:** Wufan Zhao, Michael Y. Zhu, Hemal Patel, Heather E. Whitson, Kim G. Johnson, Dilraj S. Grewal, Sharon Fekrat

**Affiliations:** 1 Department of Ophthalmology, Duke University School of Medicine, Durham, NC 27710, USA; wufan.zhao@duke.edu (W.Z.); m.zhu@duke.edu (M.Y.Z.); dilraj.grewal@duke.edu (D.S.G.); 2 Eye Multimodal Imaging in Neurodegenerative Disease (iMIND) Study Group, Durham, NC 27710, USA; hpatel@willseye.org (H.P.); heather.whitson@duke.edu (H.E.W.); kim.g.johnson@duke.edu (K.G.J.); 3 Wills Eye Hospital, Philadelphia, PA 19107, USA; 4 Department of Neurology, Duke University School of Medicine, Durham, NC 27710, USA; 5 Duke/UNC Alzheimer’s Disease Research Center (ADRC), Durham, NC 27710, USA; 6 Duke University Medical Center, 2351 Erwin Road, Durham, NC 27705, USA

**Keywords:** OCT, OCTA, neurodegeneration, plasma biomarkers, Alzheimer’s disease

## Abstract

**Background/Objectives**: Evaluating noninvasive, accessible ocular and blood-based biomarkers could aid in early risk stratification and disease detection during the preclinical phase of Alzheimer’s disease. This study investigates associations between plasma biomarkers of neurodegeneration and retinal structural and microvasculature parameters in cognitively normal adults with familial or genetic risk factors for Alzheimer’s disease. **Methods**: Forty-one participants underwent plasma sampling for glial fibrillary acidic protein (GFAP), neurofilament light chain (NfL), amyloid-beta42 (β42), amyloid-β42/40 ratio, and phosphorylated-tau217 (p-tau217), and also underwent optical coherence tomography (OCT) and OCT angiography (OCTA) imaging. Apolipoprotein E genotyping and family history of Alzheimer’s disease were recorded. Generalized estimating equations adjusting for age, sex, race, treated hypertension, Alzheimer’s disease family history, and APOE ε4 carrier status assessed associations between plasma biomarker concentrations and OCT and OCTA measurements. **Results**: Higher plasma GFAP and NfL concentrations were significantly associated with reduced superficial capillary plexus perfusion density and vessel density on macular OCTA. In additional analyses restricted to participants with plasma biomarker and retinal imaging assessments obtained within 9 months, associations with GFAP remained significant, whereas NfL associations no longer remained significant after correction for multiple comparisons. **Conclusions**: Retinal OCTA and OCT metrics may reflect general neurovascular aging in cognitively normal individuals with familial or genetic risk factors for Alzheimer’s disease.

## 1. Introduction

As the global population ages, Alzheimer’s disease (AD) is becoming a greater public health challenge, affecting approximately 7.2 million Americans aged 65 and older and an estimated 416 million individuals across the AD continuum worldwide [1,2,3]. AD is characterized by amyloid-beta (Aβ) plaque accumulation, tau pathology, and neurodegeneration that begins years to decades before the onset of cognitive symptoms [4]. During this prodromal phase, individuals may be cognitively unimpaired despite neuropathological changes in the brain [4].

AD typically presents as mild cognitive impairment (MCI) and subsequently progresses to dementia [5]. However, these clinical symptoms represent later stages along the AD continuum [6]. A high priority is the identification of reliable biomarkers to detect neurodegenerative and neuroinflammatory processes in asymptomatic individuals who are in the earliest phases of this continuum [7]. The retina is an embryologic extension of the brain and offers an opportunity to directly and noninvasively visualize neurodegenerative changes [8,9]. Compared to traditional neuroimaging modalities such as magnetic resonance imaging (MRI) or positron emission tomography (PET), optical coherence tomography (OCT) and OCT angiography (OCTA) offer micrometer-scale spatial resolution, rapid and cost-effective acquisition, and widespread clinical accessibility without the need for radiotracers [10]. Previous macular OCT and OCTA imaging studies have reported declines in superficial capillary plexus (SCP) retinal perfusion density (PD) and vessel density (VD) in individuals diagnosed with MCI and AD [11,12].

Blood-based biomarkers have more recently emerged as promising tools for the early detection of central nervous system injury [13,14,15]. For example, concentrations of plasma neurofilament light chain (NfL), a marker of axonal injury, and glial fibrillary acidic protein (GFAP), a marker of reactive astrogliosis, are elevated in MCI and AD and correlate with disease severity and progression [16,17,18]. OCT studies have further shown that thinning of the retinal nerve fiber layer (RNFL) and the ganglion cell–inner plexiform layer (GC-IPL) is associated with increased plasma NfL, GFAP, and phosphorylated-tau217 (p-tau217) concentrations as well as cognitive decline [19,20,21,22]. Furthermore, plasma concentrations of amyloid-β42 (Aβ42) and amyloid-β42/40 ratio (Aβ42/40) have been negatively correlated with RNFL thickness in the literature [23]. However, the relationship between plasma biomarkers and OCTA retinal microvascular parameters remains poorly defined [12]. Improved understanding of this relationship may help contextualize both retinal structural imaging findings and serologic metrics, and may aid in early detection during the preclinical phase of disease.

This study examines associations between plasma biomarkers of neurodegeneration, including NfL, GFAP, Aβ42, Aβ42/40, and p-tau217, as well as OCT and OCTA parameters, in cognitively normal adults who have risk factors for AD, including apolipoprotein E (APOE) ε4 carrier status and family history of AD. Characterizing these relationships provides an important baseline for identifying retinal microvascular changes that are potentially driven by early neurodegenerative processes.

## 2. Materials and Methods

This study (NCT03233646) was approved by the Duke University Health System Institutional Review Board (Pro00111831). Written informed consent was obtained from all participants and/or their designated legally authorized representative prior to study enrollment. This study adhered to the tenets of the Declaration of Helsinki regarding the enrollment of human subjects.

### 2.1. Study Population

Participants were enrolled in the Eye Multimodal Imaging in Neurodegenerative Disease (iMIND) Study between 1 January 2022 and 1 January 2025 from the Duke and UNC Alzheimer’s Disease Research Center (ADRC). This analysis included all individuals co-enrolled in Duke/UNC ADRC and iMIND during this period who were assigned a cognitive status of “unimpaired” by the ADRC diagnostic consensus panel. Exclusion criteria included history of diabetes, uncontrolled hypertension, glaucoma, and retinal or optic nerve pathology that could confound OCT or OCTA measurements. Participants with corrected distance Snellen visual acuity (VA) < 20/40 or a refractive error ±6 diopters or more were excluded. Patient interview and chart review were used to collect patient medical and ocular history. Specific clinical glaucoma screening metrics, including intraocular pressure (IOP), cup-to-disk ratio, and family history of glaucoma, were not recorded as part of this study protocol. Ultra-widefield scanning laser ophthalmoscopy (California, Optos, Marlborough, MA, USA) was performed to evaluate for vitreoretinal pathology that would exclude participants from the study. These pathologies include age-related macular degeneration, diabetic retinopathy, retinal vein or artery occlusions, epiretinal membranes, macular holes, vitreomacular traction, high myopia-related chorioretinal degeneration, and peripheral retinal tears or detachments. Participants underwent cognitive function evaluation by trained study staff using the Mini mental state examination (MMSE). Subject age, sex, race, smoking history, hypertension history, family history of neurocognitive disease, and years of education were recorded.

### 2.2. APOE Status Determination

All participants underwent APOE genotyping as part of the standardized ADRC protocol. Whole blood (3 mL per participant) was collected in vacutainer tubes containing ethylenediaminetetraacetic acid anticoagulants and arrived at the lab at room temperature. The buffy coat was separated via centrifugation and stored at −80 °C until analysis. DNA was then extracted from the buffy coat and underwent TaqMan allelic discrimination assays (Applied Biosystems, Foster City, CA, USA) which detect the APOE single gene nucleotide polymorphisms of ε2, ε3, and ε4.

### 2.3. Plasma Sample Collection

Whole blood was collected in 10 mL vacutainer tubes (total of 60 mL per participant) containing ethylenediaminetetraacetic acid anticoagulant and arrived at the lab at room temperature. Samples were centrifuged at 2000× *g* for 10 min at 4 °C. Plasma was then transferred to a 15 mL conical tube and again centrifuged under the same conditions. Plasma was then separated into 1.5 mL aliquots in 2.0 mL micronic cryotubes and stored at −80 °C until analysis. The aliquots were shipped on dry ice to the National Centralized Repository for Alzheimer’s Disease and Related Dementia (NCRAD). Plasma samples were analyzed by the single molecular array (Simoa) HD-X Analyzer (Quanterix Corp., Billerica, MA, USA). Concentrations of plasma GFAP, NfL, p-tau217, Aβ42, and Aβ42/40 were obtained.

### 2.4. OCT and OCTA Imaging

All OCT and OCTA imaging was obtained through undilated pupils within one year of the plasma collection date. Study participants were prospectively imaged using the Cirrus high-definition (HD)-5000 with AngioPlex (Carl Zeiss Meditec, Dublin, CA, USA, version 11.0.0.29946). OCT image acquisition included a 512 × 128 macular cube scan, HD enhanced depth imaging (EDI) 21-line foveal scan, and a 200 × 200 optic disk cube scan (Carl Zeiss Meditec, Dublin, CA, USA). Determination of RNFL thickness, GC-IPL thickness, and central subfield thickness (CST) was automated by AngioPlex software. A 3.46 mm diameter annulus around the optic disk was the region of interest for calculation of RNFL thickness. GC-IPL thickness was calculated from the average of 6 segments of a 60-degree, 14.13-mm^2^ ellipse around the fovea. CST was automatically calculated as the measurement between the retinal pigment epithelium and inner limiting membrane on the 512 × 128 macular cube scan.

**Figure 1:**
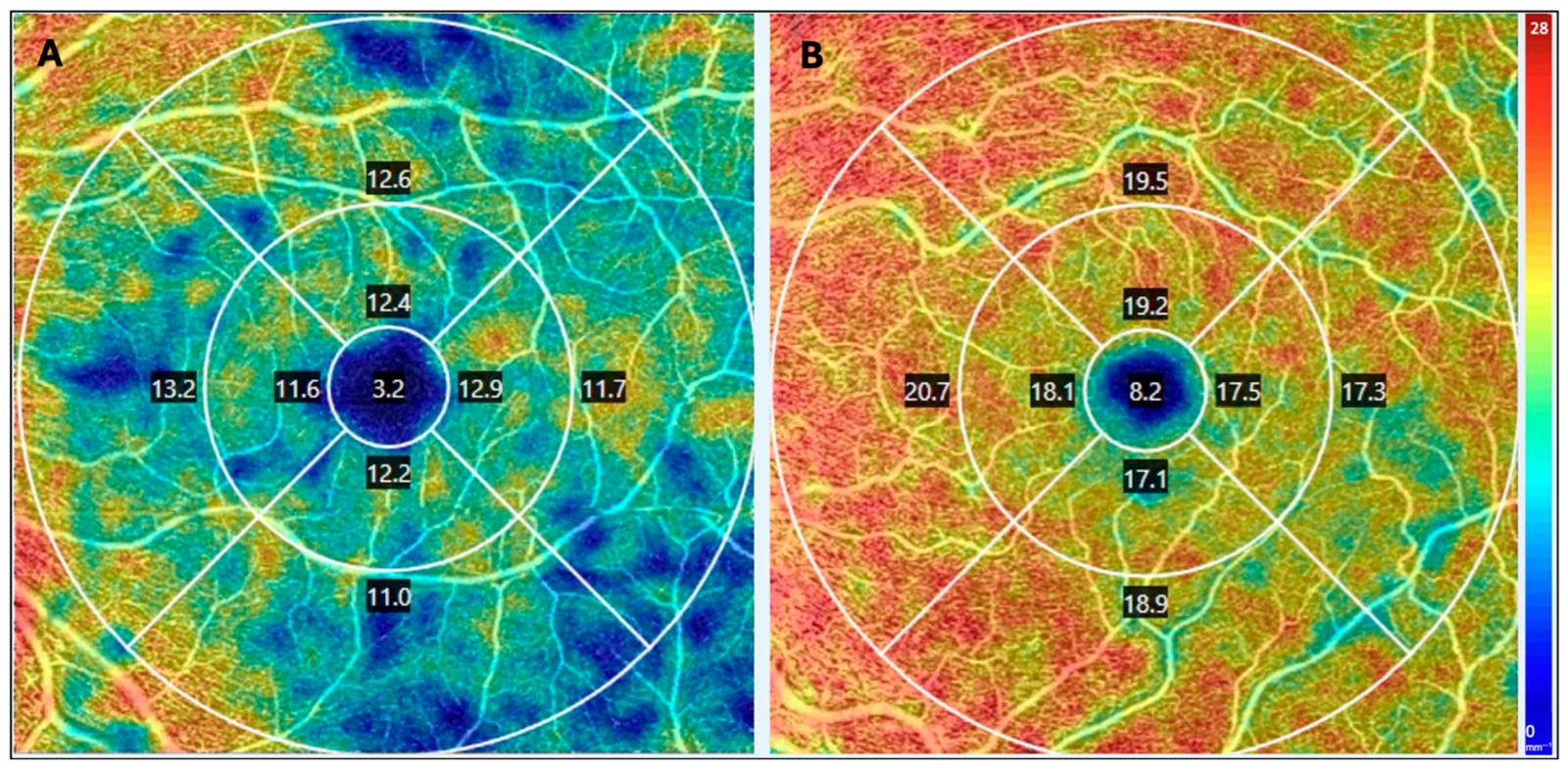
Vessel density in 6 mm OCTA scans of two cognitively normal individuals. OCTA 6 mm superficial capillary plexus vessel density is shown for an individual with GFAP of 314.3 pg/mL and NfL of 16.42 pg/mL (**A**), and for an individual with GFAP of 20.5 pg/mL and NfL of 3.29 pg/mL (**B**).

**Figure 2:**
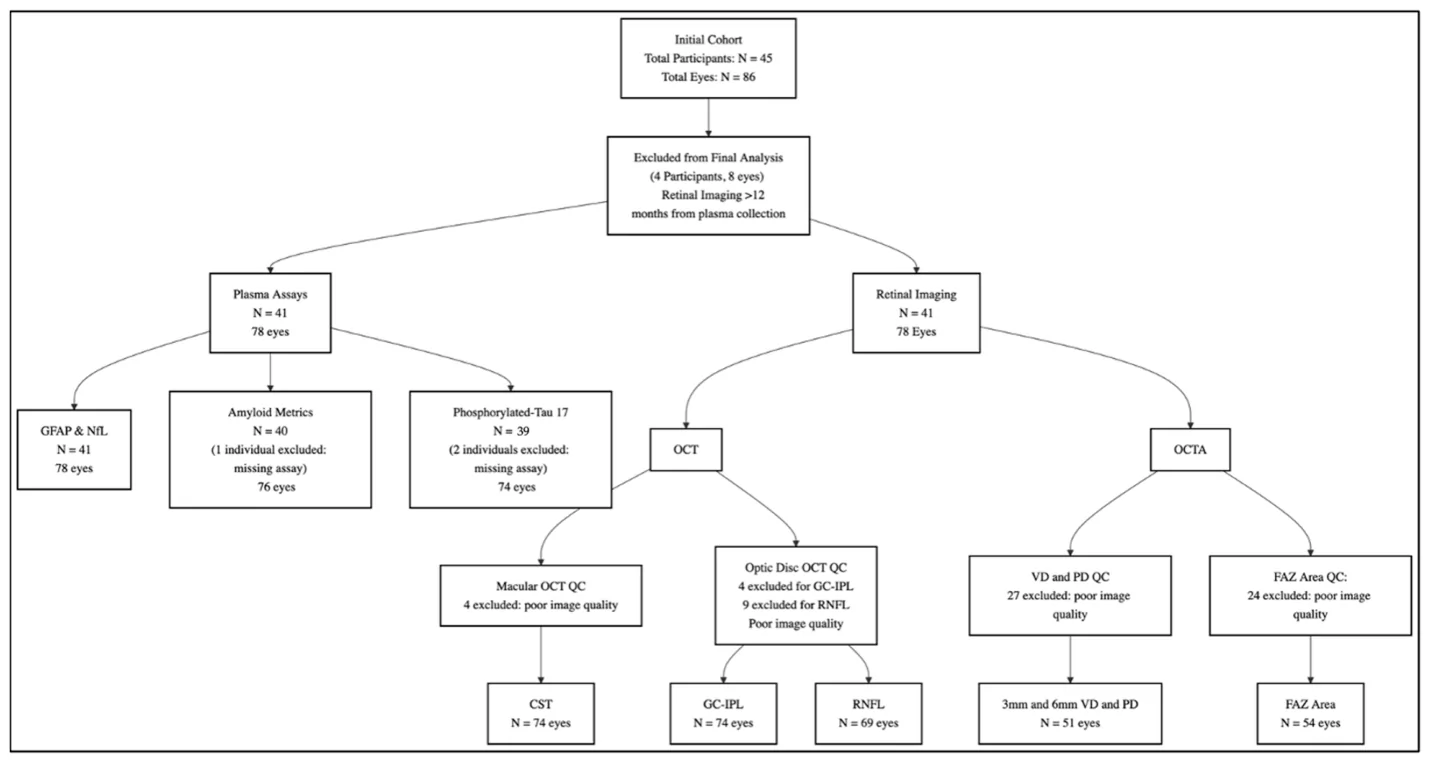
Participant and eye attrition flowchart. Participant exclusion and quality control procedures are illustrated for plasma biomarkers, OCT, and OCTA metrics. Final sample sizes for each biomarker and retinal imaging metric are shown.

OCTA images were acquired in a 3 × 3 mm and 6 × 6 mm circle centered on the fovea (Figure 1). Images with a signal strength less than 7/10, segmentation artifacts, motion artifacts, shadow artifacts, or focal signal loss were excluded from analysis (Figure 2). SCP PD and VD were calculated by AngioPlex software in the Early Treatment Diabetic Retinopathy Study (ETDRS) grid centered on the fovea in the 3 × 3 mm and 6 × 6 mm circles and rings. VD is defined as a measure of the total length of blood vessels in the region of interest (ROI) in inverse millimeters (mm^−1^). PD is defined as the area of perfused vasculature per unit area in the ROI and is unitless. The foveal avascular zone (FAZ) was automatically segmented from the 3 × 3 mm OCTA scan by AngioPlex software.

### 2.5. Statistical Analysis

Statistical analyses were performed using SAS/STAT software (SAS Institute Inc., Cary, NC, USA, version 18.5). Data distributions were evaluated using box plots. Potential outliers identified during data visualization were reviewed and, when attributable to poor OCT/OCTA image quality, the corresponding imaging measurements were excluded prior to statistical analysis. Generalized estimating equations (GEE) were used to assess associations between plasma biomarker concentrations and OCT and OCTA measurements while accounting for both eyes in the same individual. GEE models were adjusted for age, sex, race, treated hypertension, AD family history, and APOE ε4 carrier status. To account for multiple comparisons across OCT and OCTA metrics, the Benjamini–Hochberg false discovery rate (BH-FDR) was used. Statistical significance after correction was defined as a BH-FDR-adjusted *p*-value < 0.05. As a sensitivity analysis, GEE models were repeated using plasma GFAP and NfL concentrations obtained for participants who had OCT and OCTA measurements at or within 9 months of plasma collection.

## 3. Results

In this study, 86 eyes of 45 adults were imaged. Eight eyes from four separate participants were excluded due to the time interval between retinal imaging and plasma sample collection surpassing 12 months. Ultimately, 78 eyes from 41 adults were included in the final analysis. The mean age was 64.1 years (SD 8.7 years). Ten (24.4%) subjects were male, and 31 (75.6%) were female. The mean MMSE score was 29.6 (SD 0.7). Additionally, 18 subjects were APOE ε4 carriers (2 ε2/4, 13 ε3/4, 3 ε4/4) and 23 were ε4 non-carriers (3 ε2/3, 20 ε3/3). Of the 18 APOE ε4 carriers, 13 subjects had a family history of AD, and 11 subjects had a first-degree relative with AD. Of the 23 APOE ε4 non-carriers, 14 subjects had a family history of AD, and 10 subjects had a first-degree relative with AD. Of the 27 (65.8%) total subjects with a family history of AD, 21 individuals had at least one first-degree relative with AD, 9 had at least one second-degree relative, and 8 had at least one third-degree relative. Fifteen subjects had multiple relatives with AD. Nine individuals who were ε4 non-carriers (8 ε3/3, 1 ε2/3) had no family history of AD. Demographic data are presented in Table 1. Mean concentrations of plasma biomarkers and mean OCT and OCTA measurements are outlined in Table 2.

After adjusting for age, sex, race, treated hypertension, AD family history, and APOE ε4 carrier status using GEE models, increased plasma GFAP concentration was associated with decreased retinal PD and VD, particularly within the 6 mm ETDRS grid (Table 3). Increased GFAP concentrations were associated with decreased PD in the 6 mm circle (β = −0.000321, 95% CI = −0.000530 to −0.000111, *p* = 0.003, BH-FDR *p* = 0.021), 6 mm outer ring (β = −0.000338, 95% CI = −0.000558 to −0.000118, *p* = 0.003, BH-FDR *p* = 0.021), and 6 mm inner ring (β = −0.000275, 95% CI = −0.000503 to −0.000048, *p* = 0.018, BH-FDR *p* = 0.049). Increased GFAP concentrations were also associated with decreased VD in the 6 mm circle (β = −0.01169, 95% CI = −0.02003 to −0.00335, *p* = 0.006, BH-FDR *p* = 0.021), 6 mm outer ring (β = −0.011996, 95% CI = −0.02062 to −0.00337, *p* = 0.006, BH-FDR *p* = 0.021), and 6 mm inner ring (β = −0.01089, 95% CI = −0.02011 to −0.00167, *p* = 0.021, BH-FDR *p* = 0.049). Associations between increased plasma GFAP and decreased PD in the 3 mm ring (β = −0.000168, *p* = 0.033), VD in the 3 mm ring (β = −0.01036, *p* = 0.032), and the 3 mm circle (β = −0.00939, *p* = 0.049) were significant prior to multiple comparisons correction but did not remain significant after BH-FDR adjustment.

After adjusting for age, sex, race, treated hypertension, AD family history, and APOE ε4 carrier status using GEE models, increased plasma NfL concentration was associated with decreased retinal PD and VD in both the 3 mm and 6 mm ETDRS grids (Table 4). Specifically, increased NfL concentrations were associated with decreased PD in the 3 mm circle (β = −0.001665, 95% CI = −0.002721 to −0.000609, *p* = 0.002, BH-FDR *p* = 0.009), 3 mm ring (β = −0.001851, 95% CI = −0.002864 to −0.000837, *p* < 0.001, BH-FDR *p* = 0.005), 6 mm circle (β = −0.002084, 95% CI = −0.003659 to −0.000510, *p* = 0.009, BH-FDR *p* = 0.025), and 6 mm outer ring (β = −0.002158, 95% CI = −0.003818 to −0.000498, *p* = 0.011, BH-FDR *p* = 0.025). Increased NfL concentrations were also associated with decreased VD in the 3 mm ring (β = −0.1057, 95% CI = −0.1712 to −0.0402, *p* = 0.002, BH-FDR *p* = 0.009), 3 mm circle (β = −0.0960, 95% CI = −0.1619 to −0.0301, *p* = 0.004, BH-FDR *p* = 0.014), 6 mm circle (β = −0.0781, 95% CI = −0.1401 to −0.0161, *p* = 0.014, BH-FDR *p* = 0.025), and 6 mm outer ring (β = −0.0806, 95% CI = −0.1444 to −0.0167, *p* = 0.013, BH-FDR *p* = 0.025). Increased plasma NfL was additionally associated with decreased PD in the 6 mm inner ring (β = −0.001871, 95% CI = −0.003609 to −0.000133, *p* = 0.035, BH-FDR *p* = 0.054), although this association did not remain significant after multiple comparisons correction.

Plasma p-tau217 concentration was not significantly associated with any OCT or OCTA parameter in GEE models (Table 5). The plasma Aβ42/40 ratio and Aβ42 concentration were not significantly associated with any OCT or OCTA parameter after adjustment for age, sex, race, treated hypertension, AD family history, and APOE ε4 carrier status in GEE models (Table 6 and Table 7).

Nine-month sensitivity GEE analyses were performed for plasma GFAP and NfL concentrations. Increased plasma GFAP concentrations were associated with decreased PD and VD within the 6 mm ETDRS grid (Table 8). These associations remained significant after BH-FDR correction for PD in the 6 mm circle (β = −0.000325, 95% CI = −0.00052 to −0.00013, *p* = 0.001, BH-FDR *p* = 0.007) and 6 mm outer ring (β = −0.000352, 95% CI = −0.00055 to −0.00015, *p* = 0.001, BH-FDR *p* = 0.007), as well as for VD in the 6 mm circle (β = −0.01180, 95% CI = −0.01959 to −0.00400, *p* = 0.003, BH-FDR *p* = 0.011) and 6 mm outer ring (β = −0.01241, 95% CI = −0.02018 to −0.00465, *p* = 0.002, BH-FDR *p* = 0.009). Increased plasma NfL concentrations were associated with decreased PD and VD across both the 3 mm and 6 mm ETDRS grids before correction for multiple comparisons (Table 9). None of these associations remained statistically significant after BH-FDR adjustment. No significant associations were observed between either biomarker and GC-IPL thickness, RNFL thickness, or FAZ area.

## 4. Discussion

This study investigated associations between plasma biomarkers of neurodegeneration and retinal OCT and OCTA parameters in cognitively normal adults with genetic or familial risk factors for AD. Higher plasma concentrations of GFAP and NfL were significantly associated with decreased SCP PD and VD in this cohort after controlling for age, sex, race, treated hypertension, family history of Alzheimer’s disease, and APOE ε4 carrier status. Plasma p-tau217, Aβ42/40 ratio, and Aβ42 were not significantly associated with any OCT or OCTA metrics.

APOE ε4 carrier status and a family history of AD both confer a strong risk for eventual development of AD, warranting further research. Multiple studies have reported that APOE ε4 carriers have a higher cerebral amyloid burden than non-carriers [24,25]. Additionally, individuals with a family history of AD have significantly greater risk of developing AD than those without [26,27,28]. Cannon-Albright and colleagues reported that those with affected first-degree relatives had a significantly increased risk of AD, and even in the absence of affected first-degree relatives, those with affected second- or third-degree relatives also had a significantly elevated risk [29]. This increased risk may in part be due to polygenic contributions leading to neuroinflammation, dysregulation of lipid metabolism, and impaired cerebral amyloid clearance [30,31,32]. In this cohort, 18 individuals were APOE ε4 carriers and 27 individuals had a family history of AD, which may confer increased risk. Epidemiological studies indicate that heterozygous APOE ε4 carriers face a 2- to 4-fold increased lifetime risk of developing AD, while homozygous carriers face an 8- to 12-fold increased risk compared to non-carriers [33]. Similarly, individuals with at least one first-degree relative with AD have an estimated 2-fold increased lifetime risk of developing dementia [34]. Early neuroinflammatory processes may occur prior to cognitive decline even in a cognitively normal cohort with genetic and familial risk factors.

In this study, reduced SCP PD and VD were significantly associated with increased plasma GFAP and NfL concentrations. After correction for multiple comparisons, increased GFAP concentrations remained significantly associated with decreased PD and VD within the 6 mm ETDRS grid. Similarly, increased NfL concentrations remained significantly associated with decreased PD and VD in both the 3 mm and 6 mm ETDRS grids. The physiological link between reduced retinal microvascular PD and VD and elevated GFAP and NfL plasma concentrations is likely multifactorial. Previous studies in adults with AD and MCI have reported retinal microvascular changes, including pericyte loss, amyloid deposits adjacent to endothelial cells and within pericytes, as well as decreased VD and PD [35,36,37,38,39]. Because pericytes are regulators of capillary stability, vascular tone, and blood-retinal barrier integrity, their degeneration may impair retinal capillary autoregulation and contribute to reductions in retinal perfusion [39]. These findings support the hypothesis that early retinal microvascular dysfunction may precede or accompany neuronal injury during AD progression [40]. Beyond structural vascular loss, these observations are consistent with dysfunction of the retinal neurovascular unit, composed of neurons, astrocytes, Müller cells, endothelial cells, and pericytes, which collectively regulate retinal blood flow in response to local metabolic demand [41]. Experimental studies have shown that pericyte degeneration impairs neurovascular coupling, reducing capillary dilation and oxygen delivery [42]. Similar OCTA changes have also been described in APOE ε4 carriers and in those with a family history of AD [43,44]. Additionally, thinning of the GC-IPL and RNFL, reflecting neural cell loss, has been observed in individuals with AD and MCI, as well as in APOE ε4 positive cognitively normal individuals [45,46]. Dropout of neural retinal cells may also reduce retinal metabolic demand, which, in turn, could lead to decreased retinal perfusion [47,48,49,50]. These findings may indicate that elevated plasma biomarkers and retinal OCTA abnormalities represent complementary manifestations of the same underlying neurovascular disease process. Reactive astrocytosis, neuroinflammation, and axonal injury may occur concurrently with retinal microvascular dysfunction as AD pathology disrupts the neurovascular unit. Because this study is cross-sectional, the temporal relationship between retinal microvascular alterations and plasma biomarker elevations cannot be established. Nevertheless, these findings support the hypothesis that retinal OCTA metrics and plasma biomarkers reflect complementary manifestations of early AD-related neurovascular dysfunction.

Over the past decade, multimodal retinal imaging metrics have emerged as promising correlates of neurodegenerative disease [11]. For example, OCTA studies have reported increased FAZ area and decreased SCP PD and VD in the retinas of individuals with AD [51,52,53]. Decreases in SCP PD and VD on OCTA have been significantly correlated with lateral ventricle volume expansion in brain MRI of patients with AD and amnestic MCI [54,55]. Growing efforts have also focused on validating plasma biomarkers for detecting and staging AD pathology [56,57]. Bermudez and colleagues suggested that a panel consisting of plasma GFAP, NfL, Aβ42/40, and p-tau181 was useful for predicting AD-related neuropathological change [58]. The group also found that plasma GFAP and p-tau181 were most specific for predicting the anatomical progression of tau pathology through Braak staging, and that plasma Aβ42/40 and NfL were most useful for predicting amyloid plaque burden. Kivisakk and investigators found that individuals with MCI who progressed to dementia over four years had significantly higher baseline plasma concentrations of p-tau181, NfL, and GFAP compared to non-progressors, with p-tau181 and GFAP yielding strong prognostic discrimination [59]. This research adds to the literature by describing novel relationships between plasma biomarkers and retinal imaging metrics in a cognitively normal population with underlying risk factors, though the sizes of associations may be too small to serve as isolated clinical cutoffs. Combining plasma panels with OCT and OCTA could provide clinicians and researchers with new tools to identify, stratify, and monitor individuals who are at risk for AD before cognitive symptoms appear.

Additional analyses were performed for participants who obtained OCT and OCTA imaging within 9 months of plasma biomarker collection. Associations between plasma GFAP concentration and decreased 6 mm OCTA metrics remained significant after BH-FDR correction. Associations between plasma NfL concentration and decreased PD and VD no longer met the significance threshold following BH-FDR correction. Yakoub and colleagues found that in the pre-clinical period in cognitively normal individuals, markers of neuroinflammation and neurodegeneration including GFAP and NfL increase gradually over time rather than abruptly [60]. In addition, amyloid accumulates at slow rates; in an amyloid PET study involving a cognitively normal population, Bollack and investigators showed that amyloid accumulates slowly at an estimated 3.7 centiloids per year [61]. Even though markers of neuroinflammation and neurodegeneration increase slowly and gradually, OCT and OCTA imaging at variable time lengths up to 12 months from the measurement of biomarkers represents a limitation of the study. Future studies designed to validate these findings would ideally obtain retinal imaging and blood samples within 30 days to minimize temporal variability.

This study has several limitations. The sample size was small, which limits the power of statistical analysis. Specifically, analysis based on APOE ε4 genotype was not possible due to group size limitations. OCT/OCTA imaging was taken up to 12 months before or after the plasma collection date, but microvascular and structural retinal changes during this timeframe are expected to be minimal. For example, Iafe and colleagues showed VD decline of 0.0393 mm^−1^ (0.26%) per year in healthy eyes [62]. Sato and investigators demonstrated no significant difference in PD between patients across the fourth to seventh decades of age in healthy subjects [63]. As the design was cross-sectional, causal relationships cannot be established between plasma biomarkers, OCT/OCTA metrics, and conversion to MCI or AD. Longitudinal studies may allow determination of how retinal microvascular changes and plasma biomarker trajectories evolve over time. This study lacks a low-risk cognitively normal comparison group composed of individuals who are APOE ε4 non-carriers without a family history of AD. Future longitudinal studies incorporating high-risk and low-risk cognitively normal populations are needed to determine whether the observed associations between GFAP, NfL, and retinal OCTA metrics reflect age-related physiological changes or AD-specific prodromal processes.

This study focused on cognitively normal individuals with risk factors for AD, including family history of AD and APOE ε4 carrier status. While excluding individuals with diabetes and uncontrolled hypertension was necessary to avoid confounding retinal microvascular architecture, this limits the generalizability of this study’s findings to the broader, older clinical population in whom systemic vascular comorbidities are common. Additionally, while all imaging was performed through undilated pupils and rigorously screened for segmentation and signal artifacts, acute intake of vasoactive substances (such as caffeine) was not systematically recorded. Although the models in this study adjusted for a history of treated hypertension, specific vasoactive drug mechanisms or recent caffeine consumption could transiently influence functional retinal microvascular caliber and hemodynamic perfusion metrics. Additionally, this cohort included a significantly higher proportion of females (75.6%), which reflects volunteer demographic patterns commonly observed in longitudinal aging cohorts and the higher lifetime prevalence of AD in women [64]. To mitigate potential sex-related confounding on retinal architecture or biomarker concentrations, sex was adjusted for in all multivariable statistical models. Future studies with larger cohorts could characterize the relationship of plasma biomarkers and OCT and OCTA metrics in separate cohorts of individuals with MCI, AD, or cognitively normal individuals without risk factors.

## 5. Conclusions

In this cross-sectional exploratory study of cognitively normal adults with familial or genetic risk factors for AD, higher plasma GFAP and NfL concentrations were associated with lower macular SCP PD and VD. These findings suggest that retinal OCTA and OCT metrics may reflect general neurovascular aging or systemic decline, which may or may not be accelerated by AD risk factors. Longitudinal studies with low-risk control cohorts are needed to determine whether these retinal findings are associated with subsequent cognitive decline or progression along the AD continuum.

## Figures and Tables

**Table 1:** Demographic information and clinical characteristics.

Characteristic	Value
Age	64.1 ± 8.7 (34.3–78.8)
Sex	
Male	10 (24.4%)
Female	31 (75.6%)
MMSE Score	29.6 ± 0.7 (27–30)
Treated Hypertension	
Yes	7 (17.1%)
No	34 (82.9%)
Race	
White	35 (85.3%)
Black	4 (9.8%)
Hispanic	2 (4.9%)
APOE Genotype Status	
ε23	3 (7.3%)
ε33	20 (48.8%)
ε24	2 (4.9%)
ε34	13 (31.7%)
ε44	3 (7.3%)
AD Family History	
Yes	27 (65.8%)
No	14 (34.2%)
Time Between Plasma Collection and Image Acquisition, Months	Mean 6.05; Median 6.01 Std Dev 3.35; Range 0–11.47

AD = Alzheimer’s disease; APOE = apolipoprotein E; MMSE = mini mental state examination. Continuous variables (age and MMSE score) are reported as N; mean ± SD (range).

**Table 2:** OCT, OCTA, and plasma biomarker measurements.

Parameter	N	Mean (SD)	Min	Median	Max
FAZ Area	54	0.214 (0.102)	0.001	0.213	0.505
3 mm Circle PD	51	0.348 (0.029)	0.279	0.349	0.401
3 mm Ring PD	51	0.368 (0.030)	0.300	0.366	0.423
3 mm Circle VD	51	19.16 (1.79)	15.23	19.35	22.80
3 mm Ring VD	51	20.18 (1.85)	16.29	20.24	24.04
6 mm Circle PD	51	0.412 (0.041)	0.280	0.420	0.461
6 mm Outer Ring PD	51	0.424 (0.042)	0.287	0.434	0.474
6 mm Inner Ring PD	51	0.399 (0.044)	0.286	0.415	0.457
6 mm Circle VD	51	16.94 (1.58)	11.90	17.23	19.08
6 mm Outer Ring VD	51	17.30 (1.62)	12.13	17.43	19.37
6 mm Inner Ring VD	51	16.76 (1.75)	12.25	17.29	18.99
CST (µm)	74	265.96 (22.71)	206	263.5	312
GC-IPL Thickness (µm)	74	78.88 (7.06)	47	79.5	91
RNFL Thickness (µm)	69	88.96 (10.34)	49	89.0	108
Plasma GFAP (pg/mL)	78	78.71 (59.52)	20.5	65.8	314.3
Plasma NfL (pg/mL)	78	9.56 (6.74)	1.45	8.58	42.08
Plasma p-tau217 (pg/mL)	74	0.391 (0.272)	0.12	0.275	1.18
Plasma Aβ42/40 Ratio	76	0.0859 (0.0174)	0.0647	0.0849	0.1698
Plasma Aβ42 (pg/mL)	76	22.53 (6.10)	13.32	21.66	47.43

OCT = optical coherence tomography; OCTA = optical coherence tomography angiography; SD = standard deviation; PD = perfusion density; VD = vessel density; CST = central subfield thickness; RNFL = retinal nerve fiber layer; GC-IPL = ganglion cell–inner plexiform layer; FAZ = foveal avascular zone; GFAP = glial fibrillary acidic protein; NfL = neurofilament light chain; p-tau217 = phosphorylated tau217; Aβ42 = amyloid beta-42.

**Table 3:** GEE analysis results of OCT, OCTA, and plasma GFAP concentrations.

Parameter	Estimate	95% CI	*p*-Value	BH-FDR Adjusted *p*-Value
CST	0.1208	−0.0071 to 0.2488	0.064	0.081
GC-IPL Thickness	0.0128	−0.0229 to 0.0484	0.483	0.520
RNFL Thickness	0.0282	−0.0225 to 0.0788	0.276	0.322
3 mm Circle PD	−0.000151	−0.000306 to 0.000004	0.056	0.078
3 mm Ring PD	−0.000168	−0.000322 to −0.000014	0.033 *	0.058
3 mm Ring VD	−0.01036	−0.01982 to −0.00090	0.032 *	0.058
3 mm Circle VD	−0.00939	−0.01873 to −0.00005	0.049 *	0.076
6 mm Circle PD	−0.000321	−0.000530 to −0.000111	0.003 *	0.021 *
6 mm Outer Ring PD	−0.000338	−0.000558 to −0.000118	0.003 *	0.021 *
6 mm Inner Ring PD	−0.000275	−0.000503 to −0.000048	0.018 *	0.049 *
6 mm Circle VD	−0.01169	−0.02003 to −0.00335	0.006 *	0.021 *
6 mm Outer Ring VD	−0.011996	−0.02062 to −0.00337	0.006 *	0.021 *
6 mm Inner Ring VD	−0.01089	−0.02011 to −0.00167	0.021 *	0.049 *
FAZ Area	0.000034	−0.000499 to 0.000566	0.902	0.902

* *p* value considered significant if <0.05. OCT = optical coherence tomography; OCTA = optical coherence tomography angiography; CI = confidence interval; GFAP = glial fibrillary acidic protein (plasma); CST = central subfield thickness; RNFL = retinal nerve fiber layer; GC-IPL = ganglion cell–inner plexiform layer; FAZ = foveal avascular zone; PD = perfusion density; VD = vessel density.

**Table 4:** Results of GEE analysis of OCT, OCTA, and plasma NfL concentration.

Parameter	Estimate	95% CI	*p*-Value	BH-FDR Adjusted *p*-Value
CST	0.4463	−0.7142 to 1.6067	0.451	0.526
GC-IPL Thickness	−0.1245	−0.4303 to 0.1814	0.425	0.526
RNFL Thickness	0.0929	−0.3324 to 0.5182	0.669	0.669
3 mm Circle PD	−0.001665	−0.002721 to −0.000609	0.002 *	0.009 *
3 mm Ring PD	−0.001851	−0.002864 to −0.000837	<0.001 *	0.005 *
3 mm Ring VD	−0.1057	−0.1712 to −0.0402	0.002 *	0.009 *
3 mm Circle VD	−0.0960	−0.1619 to −0.0301	0.004 *	0.014 *
6 mm Circle PD	−0.002084	−0.003659 to −0.000510	0.009 *	0.025 *
6 mm Outer Ring PD	−0.002158	−0.003818 to −0.000498	0.011 *	0.025 *
6 mm Inner Ring PD	−0.001871	−0.003609 to −0.000133	0.035 *	0.054
6 mm Circle VD	−0.0781	−0.1401 to −0.0161	0.014 *	0.025 *
6 mm Outer Ring VD	−0.0806	−0.1444 to −0.0167	0.013 *	0.025 *
6 mm Inner Ring VD	−0.0691	−0.1401 to 0.0020	0.057	0.080
FAZ Area	0.001078	−0.003091 to 0.005247	0.612	0.659

* *p* value significant as <0.05. OCT = optical coherence tomography; OCTA = optical coherence tomography angiography; CI = confidence interval; NfL = neurofilament light chain (plasma); CST = central subfield thickness; RNFL = retinal nerve fiber layer; GC-IPL = ganglion cell–inner plexiform layer; FAZ = foveal avascular zone; PD = perfusion density; VD = vessel density.

**Table 5:** Results of GEE analysis of OCT, OCTA, and plasma p-tau217 concentration.

Parameter	Estimate	95% CI	*p*-Value	BH-FDR Adjusted *p*-Value
CST	26.81	−2.85 to 56.47	0.076	0.942
GC-IPL Thickness	2.30	−6.67 to 11.27	0.615	0.957
RNFL Thickness	3.85	−8.47 to 16.17	0.541	0.947
3 mm Circle PD	−0.0014	−0.0404 to 0.0377	0.946	0.984
3 mm Ring PD	−0.0063	−0.0464 to 0.0337	0.757	0.963
3 mm Circle VD	−0.0240	−2.43 to 2.38	0.984	0.984
3 mm Ring VD	−0.2617	−2.76 to 2.24	0.837	0.977
6 mm Circle PD	−0.0287	−0.0836 to 0.0262	0.305	0.942
6 mm Inner Ring PD	−0.0360	−0.0939 to 0.0218	0.222	0.942
6 mm Outer Ring PD	−0.0270	−0.0841 to 0.0300	0.353	0.942
6 mm Circle VD	−0.9036	−3.05 to 1.24	0.409	0.942
6 mm Inner Ring VD	−1.2729	−3.61 to 1.06	0.286	0.942
6 mm Outer Ring VD	−0.8054	−2.99 to 1.38	0.471	0.942
FAZ Area	0.0258	−0.0998 to 0.1515	0.687	0.962

OCT = optical coherence tomography; OCTA = optical coherence tomography angiography; CI = confidence interval; p-tau217 = phosphorylated tau217; CST = central subfield thickness; RNFL = retinal nerve fiber layer; GC-IPL = ganglion cell–inner plexiform layer; FAZ = foveal avascular zone; PD = perfusion density; VD = vessel density.

**Table 6:** Results of GEE analysis of OCT, OCTA, and plasma Aβ42/40.

Parameter	Estimate	95% CI	*p*-Value	BH-FDR Adjusted *p*-Value
CST	189.25	−346.21 to 724.72	0.488	0.558
GC-IPL Thickness	−101.87	−239.02 to 35.29	0.145	0.558
RNFL Thickness	−84.92	−290.62 to 120.78	0.418	0.558
3 mm Circle PD	0.312	−0.627 to 1.250	0.515	0.558
3 mm Ring PD	0.315	−0.641 to 1.272	0.518	0.558
3 mm Circle VD	28.75	−28.34 to 85.83	0.324	0.558
3 mm Ring VD	29.93	−29.09 to 88.94	0.320	0.558
6 mm Circle PD	0.617	−0.949 to 2.182	0.440	0.558
6 mm Inner Ring PD	0.641	−1.019 to 2.302	0.449	0.558
6 mm Outer Ring PD	0.613	−1.033 to 2.260	0.465	0.558
6 mm Circle VD	27.32	−33.31 to 87.95	0.377	0.558
6 mm Inner Ring VD	35.59	−30.31 to 101.48	0.290	0.558
6 mm Outer Ring VD	24.96	−37.60 to 87.52	0.434	0.558
FAZ Area	−0.052	−3.228 to 3.123	0.974	0.974

OCT = optical coherence tomography; OCTA = optical coherence tomography angiography; CI = confidence interval; Aβ42/40 = amyloid beta-42/40 ratio (plasma); CST = central subfield thickness; RNFL = retinal nerve fiber layer; GC-IPL = ganglion cell–inner plexiform layer; FAZ = foveal avascular zone; PD = perfusion density; VD = vessel density.

**Table 7:** Results of GEE analysis of OCT, OCTA, and plasma Aβ42 concentration.

Parameter	Estimate	95% CI	*p*-Value	BH-FDR Adjusted *p*-Value
CST	1.446	0.199 to 2.693	0.023 *	0.288
GC-IPL Thickness	−0.280	−0.620 to 0.060	0.107	0.288
RNFL Thickness	−0.076	−0.564 to 0.412	0.760	0.760
3 mm Circle PD	−0.00122	−0.00304 to 0.00059	0.187	0.288
3 mm Ring PD	−0.00155	−0.00337 to 0.00027	0.095	0.288
3 mm Ring VD	−0.0900	−0.2052 to 0.0252	0.126	0.288
3 mm Circle VD	−0.0716	−0.1842 to 0.0409	0.212	0.288
6 mm Circle PD	−0.00167	−0.00426 to 0.00093	0.208	0.288
6 mm Inner Ring PD	−0.00123	−0.00408 to 0.00162	0.398	0.464
6 mm Outer Ring PD	−0.00188	−0.00458 to 0.00081	0.170	0.288
6 mm Circle VD	−0.0624	−0.1633 to 0.0386	0.226	0.288
6 mm Inner Ring VD	−0.0441	−0.1585 to 0.0703	0.450	0.485
6 mm Outer Ring VD	−0.0709	−0.1735 to 0.0318	0.176	0.288
FAZ Area	−0.00523	−0.01156 to 0.00110	0.105	0.288

* *p* value significant as <0.05. OCT = optical coherence tomography; OCTA = optical coherence tomography angiography; CI = confidence interval; Aβ42 = amyloid beta-42 (plasma); CST = central subfield thickness; RNFL = retinal nerve fiber layer; GC-IPL = ganglion cell–inner plexiform layer; FAZ = foveal avascular zone; PD = perfusion density; VD = vessel density.

**Table 8:** Results of GEE analysis of OCT, OCTA, and plasma GFAP concentration acquired within 9 months.

Parameter	Estimate	95% CI	*p*-Value	BH-FDR Adjusted *p*-Value
CST	0.1281	0.0022 to 0.2540	0.046 *	0.072
GC-IPL Thickness	0.0247	−0.0060 to 0.0554	0.114	0.133
RNFL Thickness	0.0300	−0.0245 to 0.0844	0.280	0.302
3 mm Circle PD	−0.000147	−0.000310 to 0.00002	0.077	0.098
3 mm Ring PD	−0.000165	−0.00033 to −0.000004	0.045 *	0.072
3 mm Ring VD	−0.01058	−0.02062 to −0.00053	0.039 *	0.072
3 mm Circle VD	−0.00947	−0.01940 to 0.00046	0.062	0.087
6 mm Circle PD	−0.000325	−0.00052 to −0.00013	0.001 *	0.007 *
6 mm Inner Ring PD	−0.000251	−0.00049 to −0.00001	0.039 *	0.072
6 mm Outer Ring PD	−0.000352	−0.00055 to −0.00015	0.001 *	0.007 *
6 mm Circle VD	−0.01180	−0.01959 to −0.00400	0.003 *	0.011 *
6 mm Inner Ring VD	−0.01015	−0.01967 to −0.00062	0.037 *	0.072
6 mm Outer Ring VD	−0.01241	−0.02018 to −0.00465	0.002 *	0.009 *
FAZ Area	−0.000073	−0.00061 to 0.00046	0.790	0.790

* *p* value significant as <0.05. OCT = optical coherence tomography; OCTA = optical coherence tomography angiography; CI = confidence interval; GFAP = glial fibrillary acidic protein (plasma); CST = central subfield thickness; RNFL = retinal nerve fiber layer; GC-IPL = ganglion cell–inner plexiform layer; FAZ = foveal avascular zone; PD = perfusion density; VD = vessel density.

**Table 9:** Results of GEE analysis of OCT, OCTA, and plasma NfL concentration acquired within 9 months.

Parameter	Estimate	95% CI	*p*-Value	BH-FDR Adjusted *p*-Value
CST	1.0389	−1.3642 to 3.4419	0.397	0.397
GC-IPL Thickness	0.3178	−0.2229 to 0.8585	0.249	0.268
RNFL Thickness	0.9088	0.0121 to 1.8055	0.047 *	0.058
3 mm Circle PD	−0.0030	−0.0056 to −0.0005	0.019 *	0.051
3 mm Ring PD	−0.0031	−0.0056 to −0.0006	0.016 *	0.051
3 mm Ring VD	−0.1926	−0.3528 to −0.0325	0.018 *	0.051
3 mm Circle VD	−0.1877	−0.3446 to −0.0309	0.019 *	0.051
6 mm Circle PD	−0.0048	−0.0087 to −0.0008	0.017 *	0.051
6 mm Inner Ring PD	−0.0049	−0.0092 to −0.0005	0.029 *	0.051
6 mm Outer Ring PD	−0.0047	−0.0088 to −0.0006	0.026 *	0.051
6 mm Circle VD	−0.1753	−0.3280 to −0.0227	0.024 *	0.051
6 mm Inner Ring VD	−0.1803	−0.3576 to −0.0030	0.046 *	0.058
6 mm Outer Ring VD	−0.1683	−0.3241 to −0.0125	0.034 *	0.053
FAZ Area	0.0088	−0.0000 to 0.0176	0.050	0.058

* *p* value significant as <0.05. OCT = optical coherence tomography; OCTA = optical coherence tomography angiography; CI = confidence interval; NfL = neurofilament light chain (plasma); CST = central subfield thickness; RNFL = retinal nerve fiber layer; GC-IPL = ganglion cell–inner plexiform layer; FAZ = foveal avascular zone; PD = perfusion density; VD = vessel density.

## Data Availability

The data presented in this study are available on request from the corresponding author due to patient confidentiality.

## Abbreviations

The following abbreviations are used in this manuscript:

AD	Alzheimer’s disease
Aβ	Amyloid-beta
MCI	Mild cognitive impairment
OCT	Optical coherence tomography
OCTA	Optical coherence tomography angiography
SCP	Superficial capillary plexus
PD	Perfusion density
VD	Vessel density
NfL	Neurofilament light chain
GFAP	Glial fibrillary acidic protein
RNFL	Retinal nerve fiber layer
GC-IPL	Ganglion cell–inner plexiform layer
p-tau217	Phosphorylated-tau217
Aβ42	Amyloid-β42
Aβ42/40	Amyloid-β42/40
APOE	Apolipoprotein E
MMSE	Mini mental state examination
CST	Central subfield thickness
EDI	Enhanced depth imaging
ETDRS	Early Treatment Diabetic Retinopathy Study
ROI	Region of interest
FAZ	Foveal avascular zone
IOP	Intraocular pressure
GEE	Generalized estimating equations
BH-FDR	Benjamini-Hochberg False Discovery Rate
